# Computer networks in radiology: An introduction

**DOI:** 10.4103/0971-3026.57219

**Published:** 2009-11

**Authors:** Rochan Pant

**Affiliations:** Department of Radiodiagnosis and Imaging, Armed Forces Medical College, Pune, India

## Introduction

Personal computers (PCs) are what most of us use to store information (data) of various kinds, from photographs of the time you visited Alaska and video recordings of your child's birthday to the article on the role of perfusion MRI in brain tumors. When we want to share data stored on a PC, we transfer the data to a removable medium such as a floppy disc or a pen drive or a compact disc and then physically transport the medium to another computer. Another way is to link up the computers in some way so that data can be sent directly from one computer to another. A system in which many computers are connected to each other for this purpose is a ‘computer network.’

Computers are at the heart of modern image processing and storage. The ability computers provide to effortlessly share and transfer images and patient data with the picture archiving and communication system has changed the practice of radiology.

This article will focus on the basics of how computer networks are created.

## Components of networking

A computer network is rather like a telephone system in some ways. To use the telephone to communicate one must:

- Have the telephones and have them linked to one another
- Know the number (address) of the telephone one wants to communicate with
- Have a common language, one that persons on both ends of the telephone line understand

Similarly, in a computer network one needs:

- A physical connection between the computers. This may be achieved with cables (wired network) or by using radio waves (wireless network)
- A way of having a unique address for each computer on the network
- Common rules for communicating (like a language) so that each computer transmits and receives the data meant for it

These are achieved by both physical devices (hardware) and software (protocols).

## Hardware

The basic hardware required are the following:

- Network interface card (NIC): This is a device that connects the PC to the network through a cable (wired card) or through radio waves (wireless card). It may be internal or may be connected to the PC through a port (external card). Each network card has a unique identity (address) assigned to it, which is called the media access control. This identifies it on the network
- Communication system for signal transfer: This may use cables or radio transmitters (wireless networks)
- Devices for linking many computers together: This includes hubs, switches and routers

## Software

Along with the hardware we need the software for running the network.

- Network client: This is the software that gives the computer access to the network. For computers using Windows^®^, this would be ‘Client for Microsoft Networks’
- Protocols: These are the rules used for exchanging data on the network. The most common protocol is the TCP/IP (Transmission Control Protocol/Internet Protocol) system
- Apart from this, the computer would also need the software (drivers) to run the specific hardware (NIC) installed in the PC

## Packet switching

This is a system where large data are divided into tiny pieces called packets so that they can be sent over a network. It is like separating a book into pages and then sending it a few pages at a time, which are then reassembled elsewhere to remake the complete book. To do this successfully, we must know which book the page belongs to, how many pages there are in all and the order of the pages.

The first step is to separate the book into groups of pages, say 50 pages per package. We put each of the 50 pages into an envelope. This task is performed sequentially so that the first 50 pages go into an envelope together and so on. We put a big number ‘1’ on the envelope so that we know these are the first 50 pages. The next 50 pages get numbered ‘2’ and so on. On each envelope, we also write how many pages it contains. Finally, we write the address of the recipient on the outside along with the sender's address. On delivery, the receiver can count the total number of envelopes, see which envelopes have pages missing and check in what order the pages have to be reassembled. If an envelope is missing, or some pages are missing in an envelope, those can be mailed again by the sender to complete the book. The particular path taken by each envelope does not matter and neither does the order in which they arrive as long as all envelopes are received.

A similar procedure is followed during packet switching in a computer network. Data is divided into small ‘bite size’ packets, a process called packetizing. Packets are then analyzed by the computer for how many ‘1’s and ‘0’s they have and this information is converted to a number called checksum, which is placed in the packet. If the checksum carried out after the data is received does not match the one sent with the packet, the receiving computer recognizes that an error has occurred and asks for the packet to be sent again. Each packet also has a sequence number that tells the recipient where in the data the packet fits (like the number outside the envelope telling us which are the first 50 pages, etc.). Thus, a complete packet has:

- The data
- The source address (telling us where it is coming from)
- The destination address (telling us where it has to go)
- The sequence number (telling us where the packet fits)
- The checksum for error checking

## Ethernet

Logical network topology is the term used to refer to the rules by which data are transmitted on the network. There are a few basic types of network topology, of which Ethernet is the one in common use today.

Any network in use today is like a busy road network, with lots of data packets moving between computers, all through the same cable. The rules of the topology prevent mixing up of data.

The only method that we will really discuss is the Ethernet. (Some of you may have noticed that the network card that is installed in most PCs using a wired connection is also called an Ethernet card.) Ethernet was originally developed by the Xerox Corporation. It was modified several times and has various rules or standards, which are published in various Institute of Electrical and Electronic Engineers (IEEE) publications.

In the Ethernet topology, the basic rules are as follows:

- All computers on the same wire listen for traffic on the wire
- They do not transmit if there is traffic
- If they find a ‘quiet time,’ the data packets are transmitted
- If they sense a mix-up of packets, which is called a ‘collision,’ they stop transmitting data for some time and transmit again during a ‘quiet time’

The ‘quiet time’ is measured in nanoseconds (10^−9^s). This set of rules is known as carrier sense multiple access/collision detection and was designed by the IEEE. Because collisions make transmission slow, and the number of collisions depends on the number of computers on the same wire (called the collision domain), network designers prefer to keep less computers on each wire.

Just as an example of a different solution to the same problem, we will mention the token ring system. This is like the system used on narrow mountain roads, where only one vehicle can travel at a time. A single token is received on entering the section and is to be handed over at the gate on leaving the section. No vehicle can pass without a token. The token ring system has a token (a special data packet) that travels around a ring-like network. When a computer wants to transmit, it waits for the token, grabs it when it comes along and then transmits. When the transmission is complete, the token is released back to the network so that other computers can grab the token and transmit. This system has the advantage of not having collisions, but as more computers are added to the ring, the wait for the token becomes longer and transmission becomes slower.

Ethernet includes physical and logical components, cables of different types and various connectors and pieces of hardware. Fiberoptic technologies are also included in the Ethernet standard.

A new system that is rapidly seeing increasing use for large networks is the asynchronous transfer mode technology that was developed for data as well as video and audio transfers. This is not used for local area networks due to its complexity, but it is used for telephone networks, for example, when providing broadband internet over telephone lines.

## Open systems interconnect model (OSI model)

This is a conceptual model that is a theoretical concept of the tasks a computer network performs. It is useful for understanding the basics of networking. The OSI envisions the network as built up of layers. A layer is a concept representing a division or task of the network.

The topmost or highest layer is the user interface, like the browser that you use to watch a video on You Tube. This is called the application layer. Examples of this layer include the hypertext transfer protocol, file transfer protocol, etc.

The application layer receives inputs from the layer below it, i.e., the presentation layer or syntax layer. The presentation layer interprets the data received from the network for the particular application (or program) in use on the computer. This is analogous to an interpreter translating the languages in a diplomatic meeting. The data received by the presentation layer is in network format and is changed by it into one that the application layer understands.

Layer 5 is the session layer, which handles the dialogue between network components. This layer decides who transmits and who listens.

Layer 4 is the transport layer. This is the TCP part of TCP/IP, which sends and receives data packets. It checks for errors and where transmission fails, the data is re-transmitted.

Layer 3 is the network layer. This layer ensures that the addresses in the data packets are correctly sorted and sent on their way. This is the IP part of TCP/IP.

Layer 2 is the data-link layer. This layer is where the logical address gets converted into the actual link with the lowest layer of the physical network components like network cards, routers, hubs, etc. This layer acts as an interface between the physical network and the layer 3 tasks. Terms like Ethernet are about this layer.

Layer 1 is the physical layer, which is the actual bits and pieces that make up the network in the physical world. This layer deals with the actual hardware. It defines the electrical and physical specifications of all the devices in use on the network.

The layers from layer 1 upwards become more abstract and come closer to what we see on the computer screen at each step. Thus, data being passed up the layers get converted to something more understandable by humans, whereas that which passes downward becomes more understood by machines.

If you, like me, dislike technical jargon, let me assure you that this information is not necessary for using your computer network. It is useful as a conceptual background to understand how the pieces come together.

## TCP/IP

The TCP/IP is the most common network protocol in use today. The Internet uses this protocol. All computers on the network are identified by an IP address. This is a series of numbers that have been agreed upon. The series of numbers is separated by a ‘.,’ as shown below:

- 192.168.0.8 or 10.1.236.88
  This is called the dotted decimal notation and, by convention, there are four sets of numbers, with a maximum of three digits in each set. The highest number usable in each is 255 (from 0 to 255, i.e., a total of 256 numbers). All the computers in a particular network must have identical numbers in the first three sets. The last set of numbers (8 or 88 in the example above) is different for each computer on the network. Certain series of numbers are available for private networks, while other series are reserved for use on the Internet or other places.

There is another set of numbers, called the subnet mask, which is unique to the specific network. All computers on a network must use the same subnet mask. For example:

IP address: 192.168.0.2

Subnet mask: 255.255.255.0

This would mean that any software or application wanting to communicate with this specific computer will have to ‘dial’ the IP address of this computer, i.e., 192.168.0.2 to find it on the network.

**Figure 1 (a,b):**
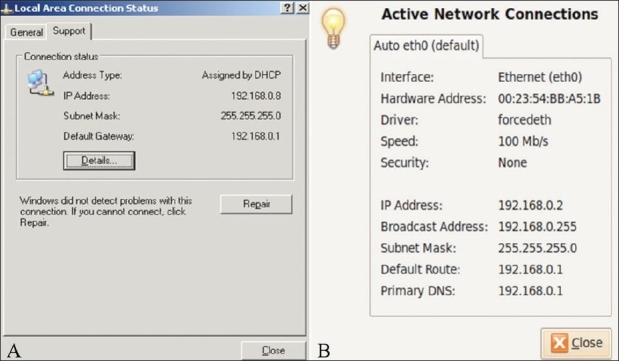
(a) Network details: Computer using Windows XP operating software (OS), (b) Network details: Computer using Linux operating software (OS).

You can find out the IP and subnet mask addresses of your own computer when it is connected to a network (like the Internet) by opening the network properties. Double click on the network icon in the bottom left hand corner of your Windows^®^ computer and click the status tab. This will show you your current IP address as well as the subnet mask [Figure 1A and B].

Because the IP addresses can be difficult to remember, the concept of domain names was introduced. This is like having a name for a telephone number stored on your mobile. You simply click on the name of the person you want to call and the number stored in the memory will be dialed. Similarly, instead of remembering an IP address, say 192.168.2.88, one could name it as ‘Suresh PC.’ This is what is done on the Internet; instead of using a complicated set of letters, you type “www.google.com” into the browser address window to communicate with a computer (server) maintained by the people at Google. This is possible because of computers called domain name servers, which maintain a list of IP addresses and corresponding domain names and redirect the request for a particular domain name to the correct computer. There frequently are multiple IP addresses associated with a domain name for busy sites like Google.

## Short note about IP addresses

These are defined by standards established by the Internet Engineering Task Force (IETF). The IETF publishes documents known as Request For Comments regarding these standards. The previous standard was the internet protocol, version 4.0 (IP, v. 4.0), which is still in use, along with the current internet protocol (IP, v. 6.0). In the IP, v. 6.0 there are 128 bits used for an address, which means that there can be 3.4 × 10^38^ unique addresses. This should suffice to keep us from running out of addresses any time soon.

**Figure 2:**
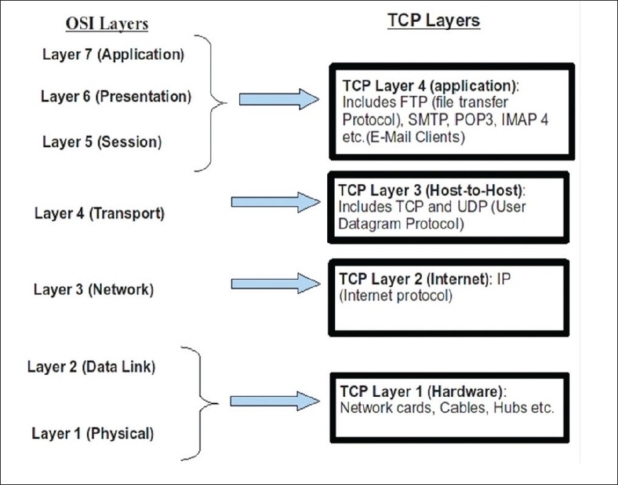
OSI Comparison with TCP/IP: The layers upwards become more abstract at each step; conversely, the layers downward becomes more concrete and physical

There is no direct correspondence between TCP/IP and OSI; however, the various layers are represented. Refer to Figure 2 for a brief explanation.

The concepts of networking and data transfer in networks discussed here are introductory and hopefully will stimulate further enquiry into this field.

